# A Touch Sensing Technique Using the Effects of Extremely Low Frequency Fields on the Human Body

**DOI:** 10.3390/s16122049

**Published:** 2016-12-02

**Authors:** Hatem Elfekey, Hany Ayad Bastawrous, Shogo Okamoto

**Affiliations:** 1 Graduate School of Engineering, Nagoya University, Nagoya 464-8603, Japan; okamoto-shogo@mech.nagoya-u.ac.jp; 2 Electrical Engineering Department, Faculty of Engineering, The British University in Egypt, Cairo 11837, Egypt; hany.bastawrous@bue.edu.eg

**Keywords:** human touch sensing, ELF electromagnetic fields, flexible touch sensing surfaces

## Abstract

Touch sensing is a fundamental approach in human-to-machine interfaces, and is currently under widespread use. Many current applications use active touch sensing technologies. Passive touch sensing technologies are, however, more adequate to implement low power or energy harvesting touch sensing interfaces. This paper presents a passive touch sensing technique based on the fact that the human body is affected by the surrounding extremely low frequency (ELF) electromagnetic fields, such as those of AC power lines. These external ELF fields induce electric potentials on the human body—because human tissues exhibit some conductivity at these frequencies—resulting in what is called AC hum. We therefore propose a passive touch sensing system that detects this hum noise when a human touch occurs, thus distinguishing between touch and non-touch events. The effectiveness of the proposed technique is validated by designing and implementing a flexible touch sensing keyboard.

## 1. Introduction

Touch sensing technologies are used in many applications, comprising smartphones and tablets, laptops, information kiosks, etc. Touch screens are very intuitive and easy to use; they also save space, because of their screen and interface spatial integration. Many touch sensing technologies have therefore been developed for commercial purposes. Examples include technologies based on infra-red sensing elements [1,2,3,4], resistive [5,6] and capacitive touch panels [7,8,9], cameras [10], the acoustic and deflection characteristics of touch panels [11,12,13], and others [14,15,16]. One of the widely used techniques is the mutual capacitive method, which is used in almost all smartphones and tablets [17]. In this method, the touch screen interface is built or constructed by rows and columns of transparent wires made from indium tin oxide. The row and column wires are separated by a thin glass layer. Each row/column is electronically charged by an individual driver circuit. When the user touches the screen at a specific position, the capacitance at the intersection between the row and the column at this position changes; the point of pressure on the panel can thus by localized by scanning all the other non-energized rows and columns and computing the capacitance at all intersections [18].

Most of the aforementioned commercially available touch sensing techniques can be classified as active sensing techniques, because touch detection depends on transmitting and receiving a signal that is perturbed by a touch. For example, in the surface acoustic wave (SAW) touch sensing, transmitters mounted on the edges of the touch screen emit sound waves that propagate through the touch screen to be received by ultrasound receivers. At the onset of touch, the human finger absorbs some of the sound waves. As a result, the signal characteristics at the receiver are altered, as the signal’s power is reduced [19]. This change in signal power is used to detect a touch. Therefore, active touch sensing techniques such as SAW use a transmitter and a receiver. On the other hand, passive sensing involves the measurement of an external signal—which is not predefined—resulting from a touch. An example of a passive touch technique is acoustic pulse recognition where sound wave receivers are used to detect a touch and no transmitters are required. Passive sensing approaches are usually low power techniques, because processing and sensing occur only at the onset of touch; in contrast, active sensing involves the continuous transmission, reception, and measurement of the detected signal, and further requires higher power levels. Some low power passive touch sensing techniques have been proposed, for which driver circuits are not required. However, some of these techniques may not be suitable to detect the touch of human fingers [20,21]. Other passive touch sensing techniques are based on piezoelectric and pyroelectric effects, as well as bending waves that propagate through the touch panel [22,23]. Nonetheless, they lack the “touch and hold” capability, which is an essential manipulation feature in touch sensing interfaces [17].

In this study, we focus on another sensing technique, called humantenna. This touch technique detects the voltage induced on the human body by nearby AC power lines. The humantenna touch sensing technique has been used for gesture recognition and as an ultralow power human body motion detector [24,25,26]. However, with the exception of our previous proposals [27,28], few passive touch sensing interfaces based on this technique have been proposed as viable alternatives to build practical devices such as keyboards or touch screens. In this paper, we propose and explore the humantenna touch technique as a touch sensing interface. The device based on this technique is passive, and requires only a signal conditioning circuit. In particular, we present a flexible touch keyboard design as an example of a low power passive touch interface based on the humantenna technique.

The rest of this paper is organized as follows. In Section 2, we introduce the operating principle of the proposed sensing technique and the associated detection scheme. In Section 3, we present the design and implementation of a keyboard based on the proposed touch sensing technique. Section 4 discusses the results of the tests made to the developed keyboard, and Section 5 concludes the paper and discusses future work.

## 2. Operation Principle

### 2.1. Effects of External Electric Fields on the Human Body

Various sources of electric and magnetic fields are present in our daily environment, such as geomagnetic sources, electrical power (AC) generation and transmission, domestic appliances, industrial equipment, telecommunications, and media broadcasting. All these sources contribute to a daily exposure to a very diverse mix of electromagnetic waves, including extremely low frequency electric and magnetic fields. Moreover, the human body’s nervous system induces the so-called biopotentials on the outer skin layer, which are in the low-frequency range [29]. The combined effect of both phenomena (external electromagnetic fields and biopotentials) creates a resultant potential on the human body that can be detected and used as a touch sensing trigger.

The voltage induced from biopotentials and the other mentioned sources of electromagnetic fields on the human body is negligible when compared with the voltage induced by the electromagnetic fields of nearby AC power lines [30]. Therefore, when developing touch sensors, the focus is placed on this latter external source of electromagnetic fields. This touch sensing technique is known as humantenna [24].

The frequency of AC power lines is very low (50/60 Hz). Therefore, a quasi-static approximation can be used, and the effects of the external electric and magnetic fields on the human body can be computed independently [31]. The magnetic permeability of the human body is similar to that of free space [32]. As a consequence, the magnetic field’s effect on the human body can be neglected, when considering indoor applications. Therefore, the electric fields produced by AC power lines are the main contributors to the voltage induced on the human body. The electric field induced by external electric fields can be obtained with the following equation:

(1) $$E_{normal,bio}=\frac{\omega_{ELF}\;\varepsilon_{o}}{\sigma_{bio}}E_{normal,air}$$

where *E_normal,bio_* and *E_normal,air_* are the normal components of the electric field in the human body and air, respectively, and *ω_ELF_*, *σ_bio_*, and *ε_o_* are the AC line frequency, whole body conductivity, and electrical permittivity of space, respectively [33]. This equation can be used to calculate the value of the induced potential on the human body when exposed to 50/60 Hz fields from typical 220/110 V AC appliances, the major sources of electric fields in common daily life.

### 2.2. AC Hum Touch Signal Acquisition and Processing

**Figure 1:**
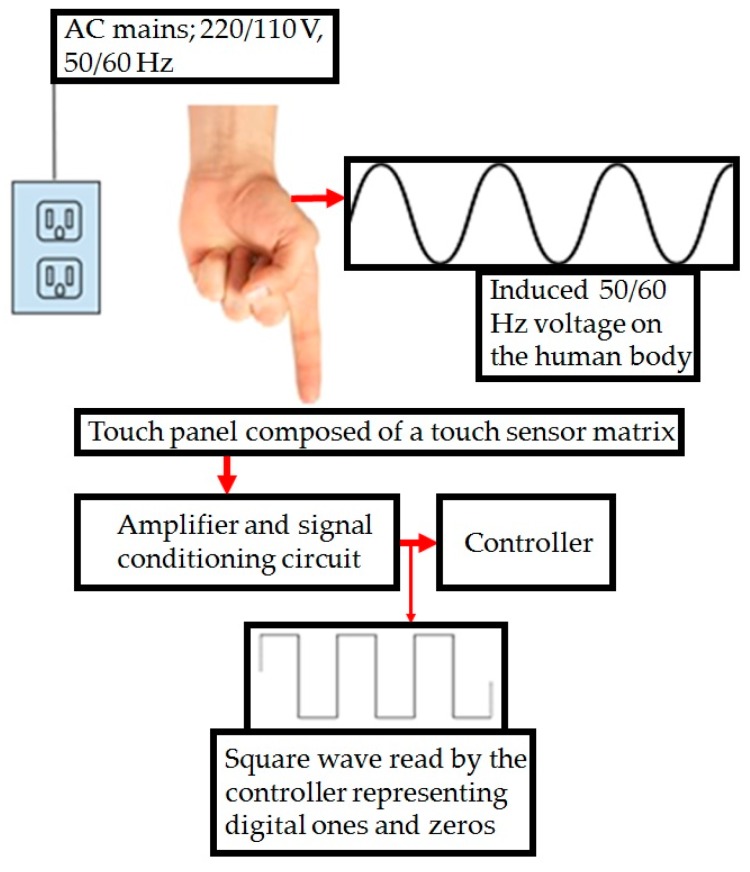
Operation principle of a touch sensing device using the humantenna approach.

A diagram of the humantenna approach as implemented here can be seen in Figure 1: a human body touches the surface of a panel, and the resulting AC signal is then amplified and conditioned for processing by a controller.

**Figure 2:**
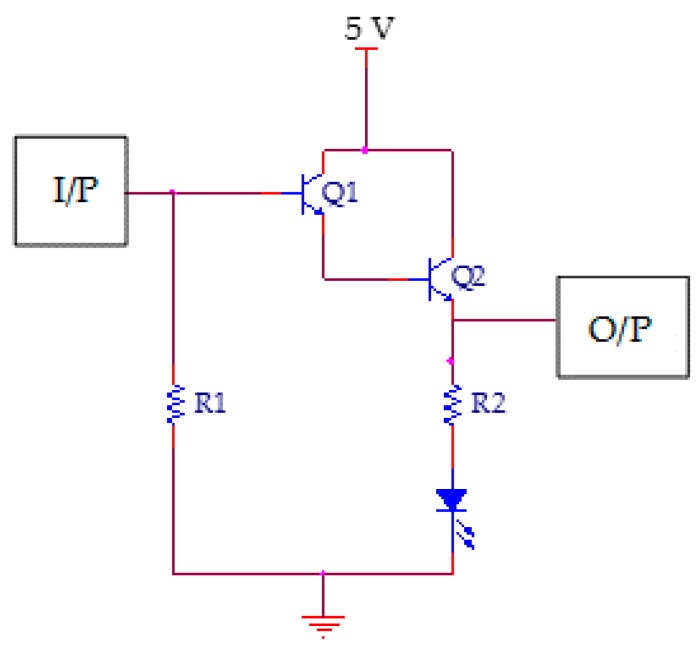
Amplifier cell schematic.

The detection circuit must be capable of processing low power sinusoidal signals. Therefore, a Darlington amplifier was used, because of its good sensitivity, linearity, and large amplification factor (typically of several hundreds or thousands A/A) [34]. The whole amplifying circuit is composed of one Darlington pair, two resistors, and an LED, as shown in Figure 2. The LED is used for indication purposes, with *R*_2_ (50 Ω) being used for LED protection. The branch containing *R*_2_ and the LED is placed in parallel with the controller input, whereas *R*_1_ (1 MΩ) is used as a pull-down resistor to nullify the parasitic effects in the circuit.

Most controllers have a small number of analogue inputs. Therefore, for convenience, digital controller inputs were used. In addition, analogue processing would imply analogue-to-digital (A/D) conversions and filtering (implying either extra modules, or a more advanced controller), whereas the use of digital inputs avoids the aforementioned needs, allowing faster and simpler processing algorithms. Therefore, leveraging the fact that the analog amplitude information is not necessary for the intended detection, both the electrical signal conditioning and the processing algorithm were designed such that the obtained signals could be connected directly to the controller’s digital input pins. Consequently, the signal delivered to the controller at each input pin is a square wave oscillating between logic zero and logic one, instead of an analogue sinusoidal wave.

**Figure 3:**
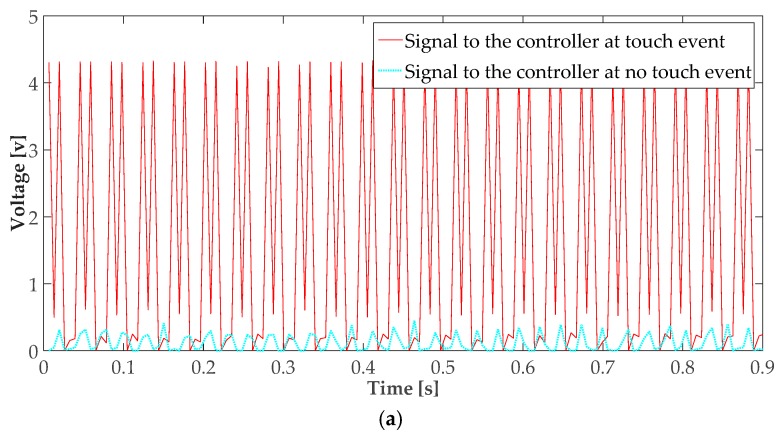

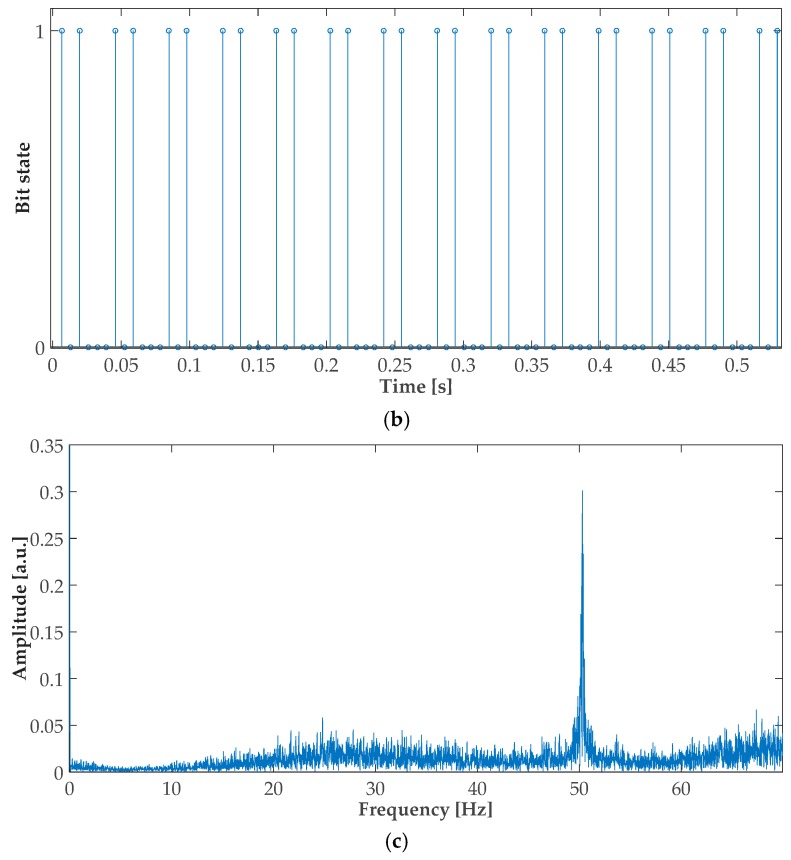
Touch signal characteristics. (**a**) Input signal to the controller before and after the touch event; (**b**) Digital signal received by the controller in the case of a touch event; (**c**) Frequency spectrum of the signal resulting from the touch event.

Figure 3a–c show the signal waveforms at the output of the amplifier in the cases of touch and no touch, the logic square wave read by the controller in the case of a touch event, and the frequency spectrum of the touch signal, respectively. As shown in Figure 3a, the input signal to the controller in the case of a touch event has a maximum voltage in excess of 4 V, whereas the maximum signal voltage in the no-touch case is below 0.5 V. For a typical controller with a supply voltage of 5 V, the maximum allowable voltage for logic zero is 1 V, whereas the threshold voltage for logic one is 3.5 V [35].

A timer is used in the controller to differentiate between touch and no-touch situations. As seen in Figure 3b, a touch-induced signal is received by the controller as a train of logic one impulses, whereas the no-touch situation corresponds to a stream of logic zero. A touch is preliminarily detected whenever a logic one is detected. When a logic zero is detected after a logic one, the timer is activated. If only logic zero is read within the specific timer duration, a final no-touch decision is made; otherwise, if further logic one is detected within the timer duration, the existence of a touch is confirmed, and a final decision (touch detection) is accordingly made. If a logic one is detected within the subsequent timer period, a “touch and hold” operation is detected. The timer duration was chosen to be 30 ms, which is a one-and-a-half period of a 50 Hz signal. Figure 3b shows that the maximum time between two consecutive ones is 26.1 ms.

## 3. Design and Implementation of a Keyboard Based on the Proposed Touch Sensing Technique

Using the proposed touch sensing technique, a touch keyboard was designed and implemented. The keyboard is comprised of a touch keypad interface, a signal conditioning circuit and a controller. A controller capable of supporting universal serial bus (USB) protocols was selected, both for convenience and to have enough digital input channels (a few tens of channels are needed in this application).

**Figure 4:**
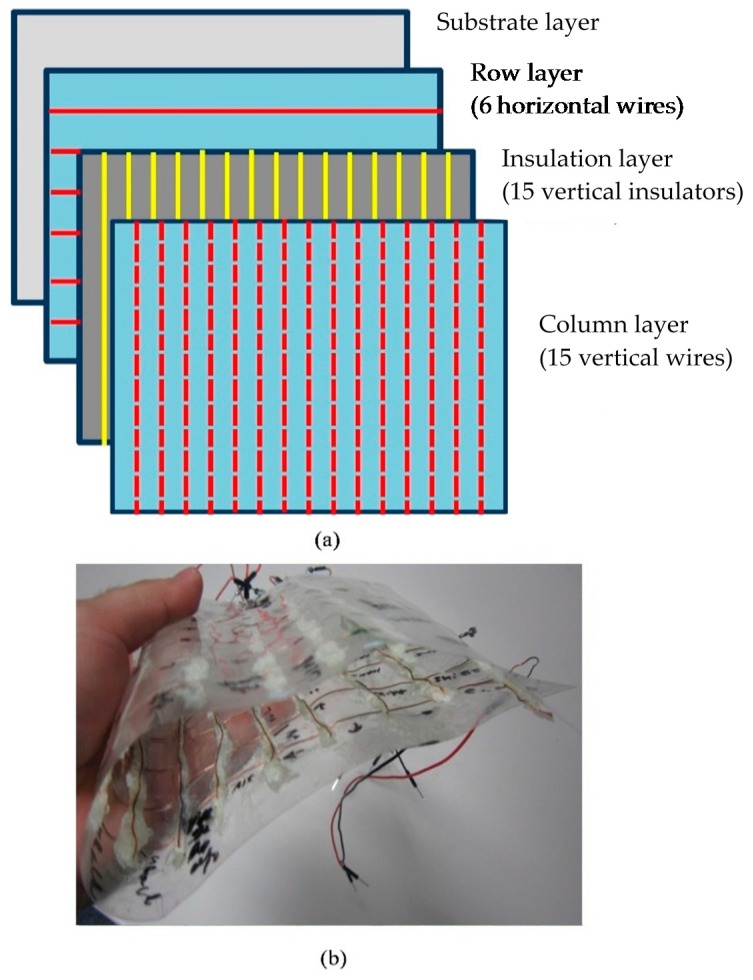
Flexible layered keyboard. (**a**) Layer structure; (**b**) Flexibility demonstration.

To allow users to directly touch the keyboard’s interface without the need for an intermediate medium, the proposed keyboard interface uses a matrix of dry electrodes [36]. The interface consists mainly of bare copper wires and insulators, which are arranged in four layers, as shown in Figure 4a. Both the copper wires and the insulator layer are 1-mm thick. The bottom transparent layer, which is referred to as the substrate layer, is a standard plastic sheet. The next layer, which is referred to as the row layer, is composed of six horizontal bare copper wires. The third layer contains 15 vertical insulators, whereas the last layer—referred to as the column layer—is composed of vertical bare copper wires, as shown by the dashed lines in Figure 4a. The construction process is comprised of three steps. First, the row layer is mounted (with equidistant rows) on the plastic substrate, and fixed in place by a cyanoacrylate adhesive. An insulator layer also made of cyanoacrylate is then mounted on top of the row layer. Finally, the column layer is mounted on the insulator layer. With this setup, the columns and rows form a matrix of coordinates. Each touch button is the intersection of a row and a column; a total of 90 distinct inputs are therefore provided. When touched by a human user, a 50 Hz signal will be induced in the corresponding row and column, thus enabling touch detection and the identification of the touched intersection.

**Figure 5:**
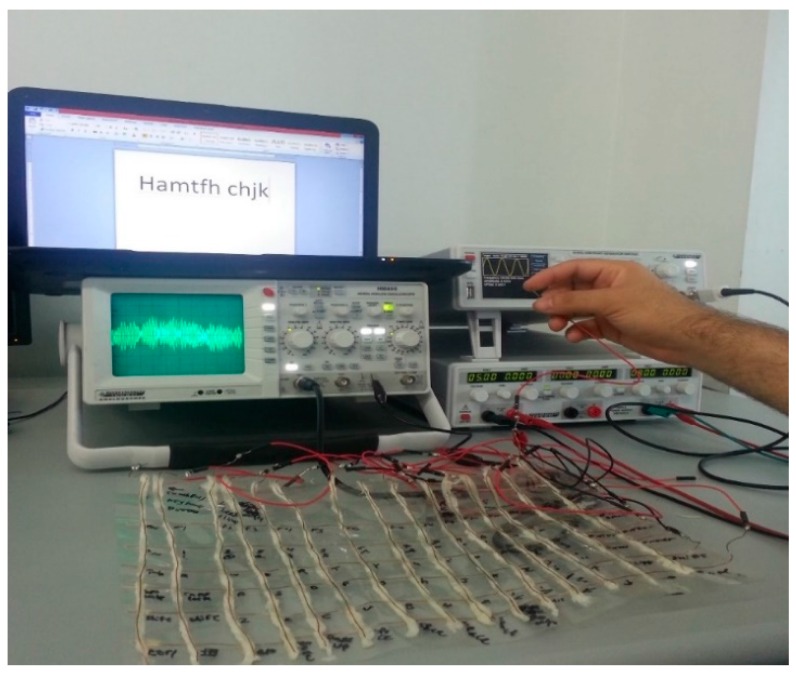
Complete keyboard structure being tested in the laboratory.

The proposed structure guarantees a unique identification of the activated touch button, because no button is represented by more than one row and one column. Moreover, this interface design has some desirable features: it is flexible and easy to store, as shown in Figure 4b; its size can easily be customized to the application; finally, it can be easily mounted on any surface, as shown in Figure 5.

The whole detection and signal conditioning circuit consisted of the 21 Darlington cells for the 21 input sensors (15 columns and 6 rows). The controller can determine the touch position by simply determining on which inputs the touch signal is present, and comparing the input pattern with a predefined look-up table. The operational algorithm is therefore very simple, and the inputs are read as standard digital signals, which is a major advantage of this keyboard implementation.

## 4. Testing the Keyboard

**Figure 6:**
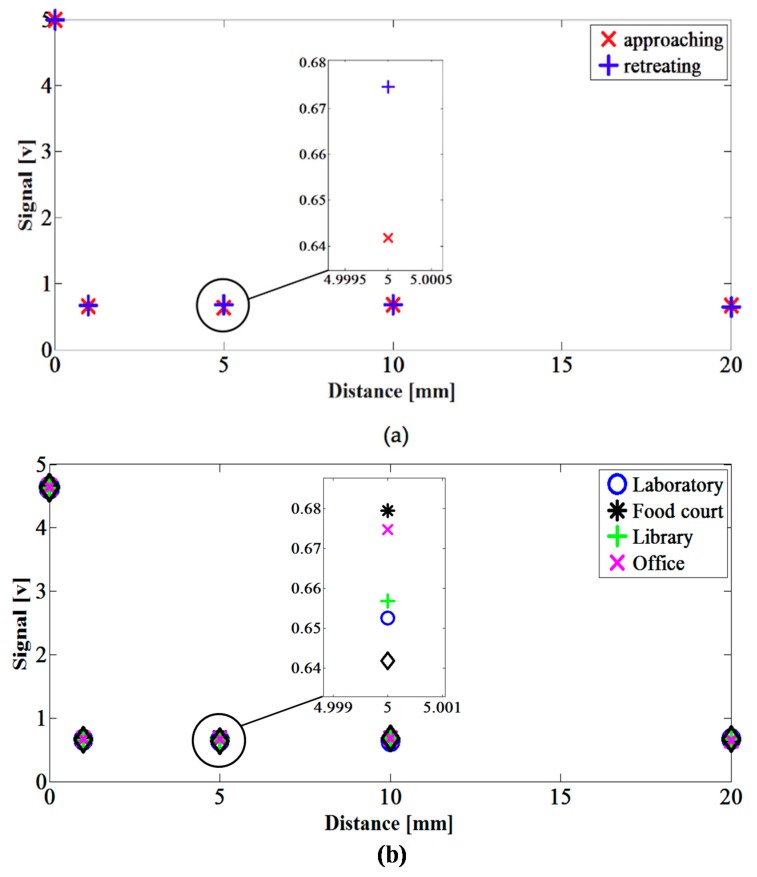
Voltage magnitude vs. distance between finger and keyboard. (**a**) Voltage detected while approaching and backing away from the keyboard; (**b**) Voltage detected in various environments.

To show the response of the proposed technique to noisy signals (which are not touch events), Figure 6a shows a comparison of the sensor voltage during a touch event with the one caused by a finger proximity at various distances from the keyboard (non-touch events). The minimum detectable voltage appeared at a distance below 0.5 mm from the keyboard interface, which indicates that the implemented keyboard is sensitive only to touch events.

To characterize the hysteresis phenomenon, Figure 6a shows the voltage readings obtained when the user’s finger is both approaching and retreating from the keyboard interface. For the implemented keyboard, the hysteresis error—the maximum difference in the recorded voltage values as the finger approached and backed away from the keyboard—was found to be less than 1%. In addition, the keyboard was tested in the various conditions and environments where its use is most expected (e.g., libraries, study rooms, residential units and laboratories). The keyboard’s sensitivity and performance showed no major variations under the different environmental conditions, as shown in Figure 6b.

The repeatability error for the implemented keyboard—defined as the maximum difference between sensor outputs recorded for the same input values, under the same conditions—was less than 6.5% for all the conducted tests, with a relative standard deviation value below 5.7%.

All the previous measurements and statistical data show the potential of the proposed touch sensing technique as a promising technology, both in terms of reliability and of cost-effectiveness, when compared to the other popular touch sensing techniques mentioned in the Introduction.

## 5. Conclusions and Future Work

In this paper, a novel touch sensing technique was proposed, based on the detection of the voltage induced on the human body by the electric fields of AC power lines.

To demonstrate the effectiveness of the proposed technique, a touch sensing keyboard was designed and implemented. The keyboard was constructed from simple components (a plastic interface, Darlington pair amplifiers, and a typical controller), and therefore did not require complex or expensive hardware. Moreover, the implemented keyboard has many desirable features: it is highly flexible, it is shock-, dust-, and water-resistant, and last but foremost it has very low implementation and operation costs. Taking into consideration that the proposed touch technique is passive and does not therefore require external generators, other possible important applications may include touch floors and safety sensors [37,38,39].

To extend our method to other contexts beyond the detection of binary events (e.g., to the localization of touch on an object surface [39]), the repeatability error mentioned above should be specifically considered. Possible causes for this error can be found in the existence of other ELF waves such as Schumann resonance [24], switched mode power supply interference noise [40], other external noises imposed by the main supply line [25], and biopotential signals [33]. In future work, the authors will investigate possible solutions to this problem, such as using a power supply with a higher-quality factor and lower total harmonic distortion (THD) to remove the power source-induced noises, applying a high quality factor band pass filter for the 48 to 62 Hz frequency band, and/or using other amplifier with lower THD and thermal noise. Furthermore, the precise energy consumption of our technique is also a topic for further investigation.

## Abbreviations

The following abbreviations are used in this manuscript:

ELF	Extremely low frequency
LED	Light emitting diode
USB	Universal serial bus
